# Safety, Dosimetry, and Feasibility of [^68^Ga]Ga-PSMA-R2 as an Imaging Agent in Patients with Biochemical Recurrence or Metastatic Prostate Cancer

**DOI:** 10.2967/jnumed.124.268318

**Published:** 2025-03

**Authors:** Liza Lindenberg, Thomas A. Hope, Frank I. Lin, Steven P. Rowe, Darko Pucar, Noella Gilbert, Daniela Chicco, Beilei He, Benedikt Feuerecker, Elena Castaldi, Lilja B. Solnes

**Affiliations:** 1 Molecular Imaging Branch, National Cancer Institute, National Institutes of Health, Bethesda, Maryland;; 2 University of California, San Francisco, California;; 3 Molecular Imaging and Therapeutics, Department of Radiology, University of North Carolina, Chapel Hill, North Carolina;; 4 Yale University, New Haven, Connecticut;; 5 Novartis Pharmaceuticals Corporation, East Hanover, New Jersey;; 6 Advanced Accelerator Applications, a Novartis Company, Turin, Italy;; 7 Novartis Pharma AG, Basel, Switzerland;; 8 Department of Radiology, Technical University of Munich, Munich, Germany; and; 9 Division of Nuclear Medicine and Molecular Imaging, The Russell H. Morgan Department of Radiology and Radiological Sciences, Johns Hopkins University School of Medicine, Baltimore, Maryland

**Keywords:** ^68^Ga-PSMA-R2, PET, dosimetry, prostate cancer, PSMA

## Abstract

Prostate-specific membrane antigen (PSMA) is highly expressed in most prostate cancers (PCs). PET and CT imaging studies using ^68^Ga-labeled PSMA ligands demonstrated the specific localization of ^68^Ga in PC lesions and distant metastatic lesions. [^68^Ga]Ga-PSMA-R2 (^68^Ga-PSMA-R2) is a PSMA-targeted PET/CT radiotracer with potential diagnostic applications. **Methods:** PROfind (NCT03490032) was a phase 1/2, open-label, multicenter study of administration of 3 MBq/kg of ^68^Ga-PSMA-R2 (from >150 to ≤250 MBq) in patients with biochemical recurrence (BCR) or metastatic PC (mPC). Participants underwent baseline conventional imaging (CT/MRI or bone scan) and PET/CT. Whole-body PET/CT imaging sequences were obtained between 20 min and 4 h after injection. Primary endpoints were safety and tolerability; secondary endpoints included biodistribution, potential lesion identification, pharmacokinetics, and dosimetry. Potential lesions were identified by 2 masked expert panels; a third panel evaluated the identified lesions. **Results:** Six patients with BCR were enrolled into phase 1, and 24 patients with BCR or mPC (*n* = 12 each) into phase 2. Thirteen treatment-emergent adverse events were reported, including 1 serious adverse event (ileus), unrelated to drug administration. All adverse events were mild or moderate and deemed not related to ^68^Ga-PSMA-R2. Peak blood concentration of ^68^Ga-PSMA-R2 was typically observed approximately 5 min after injection, steadily decreasing over 6 h. Mean absorbed radiation dose was highest in the urinary bladder wall (0.120 mGy/MBq) and kidney (0.061 mGy/MBq). No other organ mean absorbed radiation dose exceeded 0.020 mGy/MBq. Mean absorbed radiation doses in the salivary and lacrimal glands were 0.016 and 0.008 mGy/MBq, respectively. Mean total body absorbed radiation dose was 0.014 mGy/MBq. Mean effective total body dose was 0.015 mSv/MBq (range, 0.012–0.018 mSv/MBq). ^68^Ga-PSMA-R2 PET/CT detected 85 lesions in 22 participants at 1 h after injection and 103 lesions in 22 participants at 2 h after injection. Conventional imaging detected 49 lesions in 8 participants with mPC but none in participants with BCR. **Conclusion:** ^68^Ga-PSMA-R2 was well tolerated, with no drug-related treatment-emergent adverse events. Safety and preliminary imaging performance data support further development of ^68^Ga-PSMA-R2 as a diagnostic agent in patients with PC.

## Visual Abstract

**Figure:**
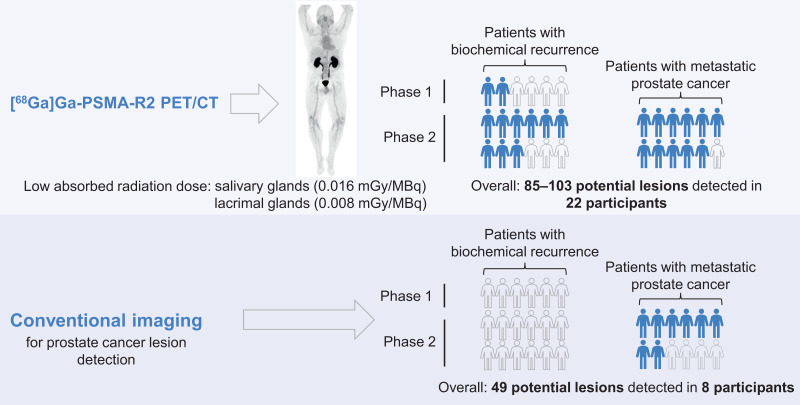

Metastatic prostate cancer (mPC) has a poor 5-y survival rate (32.3%) compared with localized disease (>99%) (1,2), underlining the importance of early detection and need for improved diagnostic and therapeutic options for mPC. Prostate-specific membrane antigen (PSMA) is weakly expressed in normal tissue but highly expressed on prostate cancer (PC) cells, correlating with tumor aggressiveness and grade (3). Therefore, PSMA is an actionable theranostic target.

Imaging for staging, restaging, and treatment selection is recommended for newly diagnosed, intermediate-risk, and high-risk PC, as well as for biochemical recurrence (BCR) and mPC (4–6). Conventional imaging technologies (MRI, CT, and bone scans) have limited sensitivity to detect early regional or mPC lesions, particularly in patients with BCR or occult mPC (7–9). PET imaging with radionuclide-labeled small-molecule PSMA ligands is highly sensitive for detection of local, regional, and mPC lesions (10). To date, the U.S. Food and Drug Administration has approved several PSMA-targeted PET imaging agents for the imaging of PSMA-positive lesions in patients with newly diagnosed or recurrent PC at risk for metastases (11–13). [^68^Ga]Ga-PSMA-11 has additionally been approved for selection of patients with PSMA-positive mPC for treatment with [^177^Lu]Lu-PSMA-617 radiopharmaceutical therapy (14).

Biodistribution of PSMA PET tracers can vary (15), but high uptake in the lacrimal and salivary glands is common. This can lead to undesirable off-target effects, such as xerostomia, when PSMA ligands are labeled with therapeutic radionuclides such as ^177^Lu or ^225^Ac (16,17). New PSMA PET tracers with favorable biodistributions, including low uptake in at-risk organs and high tumor uptake, are needed.

[^68^Ga]Ga-PSMA-R2 (^68^Ga-PSMA-R2) is a systemically delivered, PSMA-targeted PET/CT radiotracer with potential theranostic applications (18,19). PROfind was a phase 1/2 study to assess the safety, tolerability, and dosimetry of ^68^Ga-PSMA-R2 in patients with BCR or mPC. The diagnostic value of ^68^Ga-PSMA-R2 compared with conventional anatomic imaging was also assessed.

## MATERIALS AND METHODS

### Study Design

PROfind was a phase 1/2, first-in-human, open-label, single-dose study conducted at 5 sites in the United States to evaluate the primary objective of safety and tolerability of 3 MBq/kg (≥150 MBq and ≤250 MBq) of ^68^Ga-PSMA-R2. In phase 1, patients with BCR were enrolled. In phase 2, patients with either BCR or mPC were enrolled. Secondary objectives, including pharmacokinetics, biodistribution, and dosimetry of ^68^Ga-PSMA-R2, were investigated in phase 1. Imaging of PC using ^68^Ga-PSMA-R2 PET/CT versus conventional anatomic imaging was investigated in both phases.

Primary endpoints were incidence and severity of adverse events (AEs) and serious AEs and absolute changes and changes from baseline in clinical laboratory parameters, vital signs, and electrocardiograms. Secondary endpoints included absorbed radiation dose in organs and tumor lesions, pharmacokinetic parameters, SUVs, and burden and location of tumor lesions detected by ^68^Ga-PSMA-R2 compared with conventional imaging.

### Patient Populations

PROfind (NCT03490032) was conducted in accordance with the principles of the International Council for Harmonisation E6 Guidance for Good Clinical Practice, the Declaration of Helsinki, and all national, state, and local laws. Study documents were approved by the site or central Institutional Review Board. All participants provided written informed consent before entering the study.

Eligible patients were adults with histologically confirmed adenocarcinoma of the prostate and either BCR (prostate-specific antigen ≥0.2 ng/mL after radical prostatectomy or prostate-specific antigen nadir plus 2 ng/mL after radiation therapy) or mPC (castration-sensitive or castration-resistant PC with ≥1 lymph node, visceral, or bone metastasis). Supplemental Table 1 provides the complete eligibility criteria (supplemental materials are available at http://jnm.snmjournals.org).

### Interventions and Assessments

#### Preparation and Administration of ^68^Ga-PSMA-R2

Details of the preparation of ^68^Ga-PSMA-R2 are available in the supplemental materials.

**FIGURE 1.**
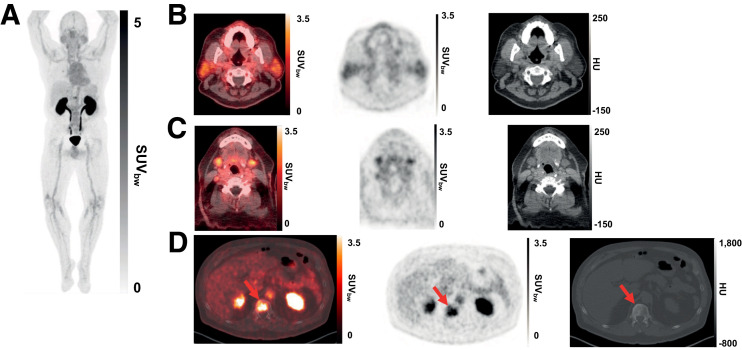
(A) Representative maximum-intensity projection and selected PET/CT images (fused, PET and CT only) of patient with mPC at 1 h after injection. (B and C) Low uptake of ^68^Ga-PSMA-R2 in salivary and submandibular glands is also demonstrated. (D) Images show heterogeneous uptake of ^68^Ga-PSMA-R2 with varying degrees of intensity in multiple spine bone lesions (vertebral sclerotic lesion with intense PSMA-R2 expression at its periphery indicated with red arrow). HU = Houndsfield unit; SUV_bw_ = body-weight SUV.

Participants received a single 3 MBq/kg intravenous injection of ^68^Ga-PSMA-R2 (≥150 MBq and ≤250 MBq total administered activity) on day 1 (Fig. 1) and were then monitored for 6 h. Safety follow-up visits were conducted on days 7 (±2 d) and 28 (±3 d).

#### Imaging

A detailed PET/CT image acquisition protocol is available in the supplemental materials.

Whole-body PET/CT imaging sequences (from the vertex to the proximal thighs) were obtained from participants in phase 1 at 20–30 min, 1 h, 2 h, and 3–4 h after ^68^Ga-PSMA-R2 injection (after urine collection; Fig. 1). On the basis of preliminary phase 1 data, participants in phase 2 underwent 2 PET/CT scans at 1 and 2 h after injection (Supplemental Fig. 1).

Imaging performance was evaluated against conventional imaging used in standard clinical practice by 3 panels of experts (supplemental materials).

Quantification of ^68^Ga radioactivity used serial PET/CT images. Visually identified focal regions of abnormal uptake of ^68^Ga-PSMA-R2 had higher SUV_max_ than background (gluteal or thigh muscle) (20). Details and other parameters recorded are available in the supplemental materials.

#### Safety

Blood and urine samples were collected, and vital signs and electrocardiograms were recorded for safety monitoring (Supplemental Fig. 1). AEs were coded using the Medical Dictionary for Regulatory Activities version 23.0 (details are in the supplemental materials).

#### Pharmacokinetics

In phase 1, blood and urine samples were collected for pharmacokinetics and dosimetry (Supplemental Fig. 1). Additional details are in the supplemental materials.

#### Dosimetry

Dosimetry analysis was performed using OLINDA/EXM version 2.0 (Hermes Medical Solutions) and calculated for organs receiving the highest dose of ^68^Ga-PSMA-R2, assessed visually from PET/CT images. Up to 10 lesions with the highest tumor-to-background ratio were analyzed per patient. Mono- and biexponential curves were fit to time–activity curves to yield time-integrated activity coefficients.

#### Baseline Conventional Imaging

CT and MR image acquisition protocols are available in the supplemental materials.

### Statistical Analyses

Sample size was based on feasibility rather than formal sample size calculation because of the early phase of the study and the use of ionizing radiation. All analyses were conducted using SAS version 9.4 or higher (SAS Institute Inc.), and the results were presented descriptively. Pharmacokinetics and dosimetry were assessed using phase 1 data. All other data are presented on the basis of the full analysis set.

Patient-level percent agreement calculations were performed to assess the level of agreement between ^68^Ga-PSMA-R2 PET/CT results and conventional imaging results (supplemental materials).

## RESULTS

### Patients

Of 32 patients screened, 30 were enrolled into the study and received ^68^Ga-PSMA-R2. Two patients without BCR or mPC were not enrolled. In phase 1, 6 patients with BCR were enrolled; in phase 2, 24 patients with BCR or mPC (*n* = 12 each) were enrolled (Supplemental Table 2). All participants completed the imaging assessment on day 1.

Most participants were White (90%, 27/30) with a median age of 70.5 y (range, 53–86 y) (Table 1). Eastern Cooperative Oncology Group Performance Status was 0 in 21 participants and at least 1 in 9 participants. The median prostate-specific antigen level was 2.05 ng/mL (range, 0.1–210.9 ng/mL) (Table 1).

**TABLE 1.** Patient Demographics and Baseline Characteristics

	Phase 1	Phase 2		
Characteristic	BCR (*n* = 6)	BCR (*n* = 12)	mPC (*n* = 12)	Overall (*n* = 30)
Age (y)	68.0 (54–76)	71.0 (55–80)	70.5 (53–86)	70.5 (53–86)
ECOG performance status				
0	6 (100.0)	9 (75.0)	6 (50.0)	21 (70.0)
≥1	0	3 (25.0)	6 (50.0)	9 (30.0)
PSA (ng/mL)	1.10 (0.5–3.5)	1.05 (0.2–21.6)	7.30 (0.1–210.9)	2.05 (0.1–210.9)
Prostate cancer history				
Time since first diagnosis (mo)	62.3 (26.0–146.0)	52.5 (7.0–232.0)	71.3 (12.0–277.0)	67.0 (7.0–277.0)
Type of castration				
Surgery	0	10 (83.3)	4 (33.3)	14 (46.7)
Pharmacologic	1 (16.7)	3 (25.0)	11 (91.7)	15 (50.0)
NA	1 (16.7)	1 (8.3)	0	2 (6.7)
Gleason score ≥6 at diagnosis				
6	0	2 (16.7)	2 (16.7)	4 (13.3)
7	4 (66.7)	8 (66.7)	3 (25.0)	15 (50.0)
8	0	1 (8.3)	3 (25.0)	4 (13.3)
9	2 (33.3)	1 (8.3)	4 (33.3)	7 (23.3)
10	0	0	0	0

Qualitative data are number and percentage. Continuous data are median and range.

ECOG = Eastern Cooperative Oncology Group; PSA = prostate-specific antigen; NA = not applicable.

### Safety and Tolerability

The mean total administered activity of ^68^Ga-PSMA-R2 was 218.95 MBq (range, 167.2–259.0 MBq). There were 13 treatment-emergent AEs in 7 participants, all classified as mild or moderate severity (Table 2). One participant with BCR experienced 1 serious AE of ileus 25 d after administration of ^68^Ga-PSMA-R2, related to the general anesthesia and pain medication administered for an elective procedure (embolization). There were no deaths and no treatment-emergent AEs related to ^68^Ga-PSMA-R2. One participant experienced a treatment-emergent AE of headache considered related to the study procedure. Fatigue (10.0%, 3/30) and rash (6.7%, 2/30) were the only AEs observed in more than 5% of participants.

**TABLE 2.** Summary of Adverse Events

	Phase 1	Phase 2		
Treatment-emergent AE	BCR (*n* = 6)	BCR (*n* = 12)	mPC (*n* = 12)	Overall (*n* = 30)
Any	1 (16.7)	4 (33.3)	2 (16.7)	7 (23.3)
Any related to ^68^Ga-PSMA-R2	0	0	0	0
Serious	0	1 (8.3)	0	1 (3.3)
Ileus	0	1 (8.3)	0	1 (3.3)
Leading to death	0	0	0	0
Leading to study discontinuation	0	0	0	0
Occurring in ≥5% patients				
Fatigue	0	2 (16.7)	1 (8.3)	3 (10.0)
Rash	0	1 (8.3)	1 (8.3)	2 (6.7)
Occurring in <5% patients				
Influenzalike illness	0	1 (8.3)	0	1 (3.3)
Pyrexia	0	1 (8.3)	0	1 (3.3)
Dysgeusia	0	1 (8.3)	0	1 (3.3)
Headache	0	0	1 (8.3)	1 (3.3)
Leukocytosis	1 (16.7)	0	0	1 (3.3)
Ileus	0	1 (8.3)	0	1 (3.3)
Dysuria	0	0	1 (8.3)	1 (3.3)
Cough	0	0	1 (8.3)	1 (3.3)

Qualitative data are number and percentage.

Changes in hematology and urinalysis were rare and not clinically significant, except for 1 event of elevated whole blood and neutrophil counts and 1 event of blood in urine; both were assessed as nonserious. No clinically significant changes in blood chemistry, vital signs, or electrocardiograms were recorded.

### Pharmacokinetic Analyses

^68^Ga-PSMA-R2 was detected in the blood 0.083–6 h after injection. Peak blood concentration was typically approximately 5 min after injection followed by a steady decrease over 6 h. Urinary excretion of ^68^Ga-PSMA-R2 over 6 h after injection varied from 44% to 81% of the total administered activity. The terminal half-life was approximately 2–4 h based on total systemic clearance of 3,730–8,330 mL/h and apparent volume of distribution of 18,300–25,200 mL.

### Dosimetry (Phase 1)

The mean absorbed radiation dose was highest in the urinary bladder wall (0.120 mGy/MBq; range, 0.048–0.198 mGy/MBq), followed by the kidney (0.061 mGy/MBq; range, 0.041–0.093 mGy/MBq). The mean absorbed radiation dose of any other organ did not exceed 0.02 mGy/MBq (Table 3). Mean absorbed radiation doses in the salivary and lacrimal glands were 0.016 mGy/MBq (range, 0.013–0.022 mGy/MBq) and 0.008 mGy/MBq (range, 0.006–0.011 mGy/MBq), respectively (Table 3; Fig. 1). The mean effective whole-body dose was 0.015 mSv/MBq (range, 0.012–0.018 mSv/MBq) (Table 3). The mean non–decay-corrected tissue activity from ^68^Ga-PSMA-R2 was higher in the liver and kidneys than it was in other organs (Supplemental Table 3). In all participants, non–decay-corrected tissue time–activity curves for the brain, heart wall, kidneys, liver, lungs, salivary glands, and spleen showed exponential decrease in percentage injected activity with time. The trend was similar for the brain in 4 participants and for the lacrimal glands in 3 participants.

**TABLE 3.** Dosimetry by Organ (Phase 1)

	Absorbed radiation dose (mGy/MBq)		
Organ or tissue	Mean	SD	Range
Urinary bladder wall	0.120	0.052	0.048–0.198
Kidneys	0.061	0.019	0.041–0.093
Adrenal glands	0.019	0.002	0.017–0.021
Heart wall	0.018	0.002	0.016–0.021
Liver	0.017	0.003	0.013–0.021
Spleen	0.017	0.004	0.013–0.023
Prostate	0.016	0.001	0.016–0.018
Salivary glands	0.016	0.003	0.013–0.022
Rectum	0.016	0.000	0.015–0.016
Gallbladder wall	0.015	0.000	0.014–0.015
Small intestine	0.015	0.000	0.014–0.015
Pancreas	0.014	0.000	0.014–0.015
Left colon	0.014	0.000	0.014–0.015
Right colon	0.014	0.000	0.014–0.015
Stomach wall	0.014	0.000	0.013–0.014
Testes	0.013	0.000	0.013–0.013
Thymus	0.013	0.000	0.012–0.013
Esophagus	0.013	0.000	0.012–0.013
Thyroid	0.013	0.000	0.012–0.013
Eyes	0.011	0.000	0.010–0.012
Osteogenic cells	0.011	0.003	0.009–0.016
Red marrow	0.011	0.000	0.010–0.011
Lacrimal glands	0.008	0.002	0.006–0.011
Lungs	0.008	0.001	0.007–0.009
Brain	0.003	0.000	0.002–0.003
Total body	0.014	0.000	0.014–0.014
Effective dose (mSv/MBq)	0.015	0.002	0.012–0.018

### Lesion Detection

At 1 h after injection, the mean lesion SUV_max_ was 5.83 and the mean lesion tumor-to-background ratio was 9.58 (Supplemental Tables 4–7). In participants positive for BCR by PET/CT at 1 h, prostate-specific antigen levels were no more than 1 ng/mL in 5 participants and greater than 1 ng/mL in 6 participants. Results were similar at 2 h after injection. Patient-level positive percent agreement was 87.5% (95% CI, 47.4–99.7%), negative percent agreement was 31.8% (95% CI, 13.9–54.9%), and overall percent agreement was 46.7% (95% CI, 28.3–65.7%).

Using PET/CT, 85 potential lesions in 22 participants were detected at 1 h after injection: 33 in participants with BCR and 52 in participants with mPC. At 2 h, 103 potential lesions in 22 participants were detected using PET/CT: 45 in participants with BCR and 58 in participants with mPC. Using conventional imaging, 49 potential lesions were detected in 8 participants with mPC but none in participants with BCR (Table 4; Supplemental Table 8).

**TABLE 4.** Summary of Patient Level Agreement of ^68^Ga-PSMA-R2 PET/CT with Conventional Imaging

Parameter	Time	Conv+	Conv−	Total
BCR (*n* = 18)				
PET+	1 h	0	11	11
	2 h	0	13	13
PET−	1 h	0	7	7
	2 h	0	5	5
mPC (*n* = 12)				
PET+	1 h	7	4	11
	2 h	7	2	9
PET−	1 h	1	0	1
	2 h	1	2	3
Total (*n* = 30)		8	22	
Overall positive percent agreement		87.5% (95% CI, 47.4–99.7%)		
Overall negative percent agreement		31.8% (95% CI, 13.9–54.9%)		
Overall percent agreement		46.7% (95% CI, 28.3–65.7%)		

Three patients with mPC did not have ^68^Ga-PSMA-R2 PET/CT scan data available at 2 h after injection.

Conv+ = positive by conventional imaging; Conv− = negative by conventional imaging; PET+ = positive by PET; PET− = negative by PET.

Overall, 7 participants were positive by both PET/CT and conventional imaging (all mPC), 15 participants were positive by PET/CT only, 1 was positive by conventional imaging only (mPC), and 7 were negative by both PET/CT and conventional imaging (Table 4).

## DISCUSSION

PROfind was a phase 1/2, first-in-human study of ^68^Ga-PSMA-R2 in 30 adults with BCR or mPC. A single dose of ^68^Ga-PSMA-R2 was generally well tolerated, with no safety concerns raised, and a safety profile consistent with other PSMA-targeted PET agents (21–23).

Absorbed radiation doses were highest in the urinary bladder wall and kidney, reflecting the urinary excretion of^ 68^Ga-PSMA-R2. These absorbed radiation doses and the mean effective whole-body absorbed radiation dose are consistent with other ^68^Ga- and ^18^F-labeled imaging agents (24–26) and with The Radioactive Drug Research Committee recommendations. Mean absorbed radiation doses in other at-risk organs were low, including in the salivary (0.016 mGy/MBq) and lacrimal (0.008 mGy/MBq) glands, which have high expression of PSMA (27) and in which high uptake of PSMA ligands has been previously observed (28). Previous studies have reported mean absorbed radiation doses of 0.089–2.1 and 0.11–3.8 mGy/MBq in the salivary and lacrimal glands, respectively (25,29–31), but direct comparisons between studies are difficult because of variations in study designs, PSMA agents, and volumes used for dose estimation. The pharmacokinetic and biodistribution profiles support the use of the PSMA-R2 scaffold as a PSMA-targeted imaging agent. The relatively low absorbed radiation doses in the lacrimal and salivary glands may also support its use in selection of patients for PSMA-targeted radiopharmaceutical therapy.

Overall, more participants with potential tumor lesions and more potential lesions were detected with ^68^Ga-PSMA-R2 PET/CT than with conventional imaging. This underlines the need to correlate conventional imaging with additional methods such as PET/CT for PC diagnosis. Potential lesions identified only by PET/CT may represent earlier-stage or smaller lesions not detectable by conventional imaging. Conversely, potential lesions identified only by conventional imaging may represent PSMA-negative tumors (27,32), sclerotic lesions that would not readily take up ^68^Ga-PSMA-R2, or scar tissue from previously treated lesions. Lesions detected only by conventional imaging were predominantly bone, and more bone lesions were detected by conventional imaging than by PET/CT. It is possible that this may be a result of persistent osteoblastic reaction at the sites of nonviable tumors from treated or inactive metastatic disease.

^68^Ga-PSMA-R2 PET/CT findings and SUV_max_ were similar at 1 and 2 h after injection. Imaging at 1 h was considered optimal because of the rapid clearance of ^68^Ga-PSMA-R2. Imaging at 2 h may be useful for designing targeted treatment plans in special cases of BCR when no lesions are identified in conventional or 1-h PET/CT images.

The biodistribution and dosimetry data of PSMA-R2 also indicate its potential use as a therapeutic agent when coupled with β-emitting (e.g., ^177^Lu) or α-emitting (e.g., ^225^Ac) radionuclides. After the VISION study, PSMA-targeted radiopharmaceutical therapy for the treatment of PC has drawn increasing attention, with several trials under way (33). Compared with other PSMA-targeting scaffolds, PSMA-R2 has the distinct advantage of having relatively low absorbed radiation doses in at-risk organs and tissues (kidney, bone marrow, and salivary and lacrimal glands) (25,34). A randomized phase 1/2 study aimed at characterizing the safety, tolerability, pharmacokinetics, and antitumor activity of [^225^Ac]Ac-PSMA-R2 is ongoing (SatisfACtion, NCT05983198) (35).

A limitation of the study is that no corroborative immunohistochemistry or other data were collected to assess PSMA positivity, so it is possible that some patients were false positives. Furthermore, no approved reference standard for PET/CT data was available at the time of the study; instead, the imaging performance was evaluated against conventional imaging used in standard clinical practice by 3 panels of experts. Although the sample size was small, we believe it was adequate to assess the study objectives and was similar to or higher than that in other published studies investigating PSMA agents in PC (25,30,31).

## CONCLUSION

The safety and imaging performance of ^68^Ga-PSMA-R2 for detecting potential PSMA-positive lesions in patients with BCR or mPC supports the use of ^68^Ga-PSMA-R2 as an imaging agent for patients with PC. Very low salivary and lacrimal absorbed radiation doses could be a particular advantage of ^68^Ga-PSMA-R2 for the selection of patients for PSMA-targeted radiopharmaceutical therapy.

## DISCLOSURE

Thomas Hope receives grant funding from Clovis Oncology, GE HealthCare, Janssen, Lantheus, Novartis, Telix Pharmaceuticals, the National Cancer Institute (R01CA235741 and R01CA212148), and the Prostate Cancer Foundation; receives personal fees from Bayer, BlueEarth Diagnostics, Cardinal Health, Lantheus, and RayzeBio; and receives fees from and has an equity interest in AdvanCell and Curium. Frank Lin has waived registration fees for attending the Society of Nuclear Medicine and Molecular Imaging annual meetings. Steven Rowe receives grants, contracts, and consulting fees and holds stock or stock options from Precision Molecular, Inc.; receives grants or contracts from PlenaryAI; and receives payment or honoraria from Lantheus Pharmaceuticals, Inc. Benedikt Feuerecker receives grants from the Bundesministerium fuer Bildung und Forschung (BMBF), Germany, was a previous consultant for and is currently employed by Novartis Pharma AG, and works part-time at Klinikum rechts der Isar, Technical University of Munich, Munich, Germany. Noella Gilbert is a current employee of Novartis. Daniela Chicco and Beilei He were former employees of Novartis and held Novartis stock options. Daniela Chicco holds patents for PSMA ligands and uses thereof under International Publication Number WO 2021/001360 A1. Lilja Solnes receives funding from Cellectar, Lantheus, Novartis, Precision Molecular Imaging, and Viewpoint; receives royalties from Elsevier; and receives consulting fees for serving on the Safety Review Board Progenics Steering Committee for Novartis. No other potential conflict of interest relevant to this article was reported.

## Notes

KEY POINTS **QUESTION:** Can ^68^Ga-PSMA-R2 be used as a PET/CT imaging agent for the detection of PC lesions? **PERTINENT FINDINGS:** In this study of 30 adults with BCR or mPC, ^68^Ga-PSMA-R2 PET/CT showed increased sensitivity for detection of potential PC lesions versus conventional imaging modalities. The safety, dosimetry, and biodistribution of ^68^Ga-PSMA-R2 was also consistent with other PSMA-targeted PET agents. **IMPLICATIONS FOR PATIENT CARE:** These findings support further clinical development of PSMA-R2 as a theranostic scaffold.
